# New Appliances and Things Medical

**Published:** 1898-02-19

**Authors:** 


### NEW APPLIANCES AND THINGS MEDICAL.

[We shall be glad to receive, at our Office, 28 & 29, Southampton Street, Strand, London, W.C., from the manufacturers, specimens of all new preparations and appliances which may be brought out from time to time.]

### LIPTON'S CONCENTRATED FLUID BEEF.

(Lipton, City Road, E.C.)

We have received from Messrs. Lipton a sample of their concentrated fluid beef. It is a well made preparation containing in an agreeable form the nutritive property of meat. Analysis shows that it differs from many of the ordinary kinds of fluid meat in containing a good proportion of meat fibrine. It contained 53·2 per cent. of meat extractives and fibrine, 15·4 per cent. mineral constituents, and 31 4 per cent. of water. When made as directed, in the proportion of a teaspoonful to a cup of boiling water, it furnishes a very appetising and nutritive cup of beef tea. Lipton's concentrated fluid beef is evidently a high-class preparation.

